# Biological effects of deuteronation: ATP synthase as an example

**DOI:** 10.1186/1742-4682-4-9

**Published:** 2007-02-22

**Authors:** Abdullah Olgun

**Affiliations:** 1 Biochemistry Laboratory, TSK Rehabilitation Center, Gulhane School of Medicine, 06800 Bilkent Ankara, Turkey

## Abstract

**Background:**

In nature, deuterium/hydrogen ratio is ~1/6600, therefore one of ~3300 water (H_2_O) molecules is deuterated (HOD + D_2_O). In body fluids the ratio of deuterons to protons is ~1/15000 because of the lower ionization constant of heavy water. The probability of deuteronation rather than protonation of Asp 61 on the subunit c of F_0 _part of ATP synthase is also ~1/15000. The contribution of deuteronation to the pKa of Asp 61 is 0.35.

**Theory and Discussion:**

In mitochondria, the release of a deuteron into the matrix side half-channel of F_0 _is likely to be slower than that of a proton. As another example, deuteronation may slow down electron transfer in the electron transport chain (ETC) by interfering with proton coupled electron transport reactions (PCET), and increase free radical production through the leakage of temporarily accumulated electrons at the downstream complexes.

**Conclusion:**

Deuteronation, as exemplified by ATP synthase and the ETC, may interfere with the conformations and functions of many macromolecules and contribute to some pathologies like heavy water toxicity and aging.

## Background

### Deuteronation

In nature, the ratio of deuterium to hydrogen is ~1/6600 [1], therefore the ratio of deuterated + heavy water (HOD + D_2_O) to water (H_2_O) is ~1/3300. In the atomic nucleus of hydrogen there is only one proton, while in deuterium there is one proton and neutron. One dissociation product of water is a proton (H^+^)/H_3_O^+^; for D_2_O, the equivalent product is a deuteron (proton + neutron) (D^+^)/H_2_OD^+ ^+ D_3_O^+^. The ionization constant of D_2_O (1.95 × 10^-15^, pD = 7.35) is 5.17-fold lower than that of H_2_O (1.008 × 10^-14^, pH = 6.99) [2]; thus, the ratio of protons in water to deuterons in heavy water is 2.27. Although the ratio of hydrogen to deuterium is 1/6600, the ratio of deuterons to protons is 1/2.27 × 6600 = ~1/15000 (Table 1). Therefore, in any biological process in which protonation is involved, there is ~1/15000 chance of deuteronation.

**Table 1:** Parameters mentioned in the text and their values

**Parameter**	**Value**
D/H	~1/6600 [1]
(HOD+D_2_O)/H_2_O	~1/3300
Deuteron/Proton	~1/15000
Proton transit velocity from F_0_	~1000/s [11]
Half life of F_0 _C subunit	40–50 hours [12]
Number of ATP synthases in mitochondrion	in liver mitochondria: ~15000 [22]

### ATP Synthase

ATP synthase (F_0_F_1 _ATPase), an inner mitochondrial membrane enzyme complex, is a molecular motor that uses protonation to generate a wheel-like rotation to catalyse the synthesis of ATP, which is the most important energy currency in living systems. During mitochondrial electron transport, protons are pumped from the matrix to the intermembrane space by the electron transport chain (ETC) complexes I, III and IV by a mechanism coupling electron transport to proton passage. The proton gradient thus formed and the consequent proton motive force rotates the rotor part of F_0. _This torque is transmitted by the stalk part of ATP synthase to F_1_, which produces one ATP with the passage of approximately three protons [3-5].

**Figure 1:**
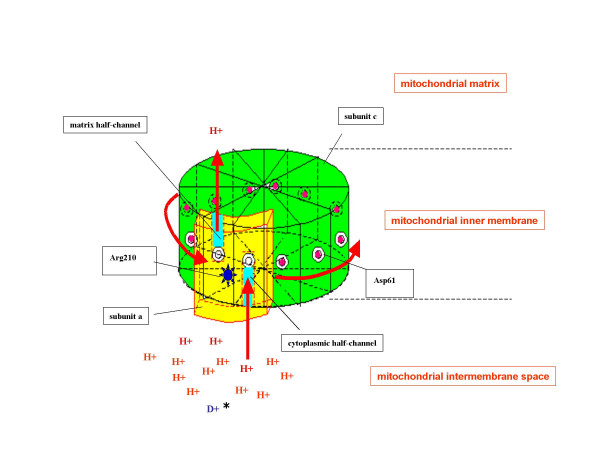
**Proton/deuteron passage from F_**0 **_of ATP synthase in mitochondria**. Protons enter the cytoplasmic half-channel and reach Asp61 on subunits c. Protonated Asp61 moves into the lipid bilayer. When protonated Asp61 reaches the matrix half-channel, it is deprotonated by the stator charge of Arg210 on subunit a. A temporary stutter of the rotor is expected during the passage of deuteron. *The ratio of deuterons (D+) to protons (H+) is ~1:15000.

F_0 _is hydrophobic, spans the mitochondrial inner membrane and is estimated to have ~10 c subunits. The c subunits form a wheel-like structure that is a part of the "rotor". In *E. coli*, there is an aspartic acid residue in the middle of the second helix of subunit c. Subunit a of F_0 _binds to the outside of the rotor and forms part of the "stator". There are two proton half-channels (termed cytosolic and matrix in mitochondria) of "subunit a", on the interface between subunits c and a. The proton concentration in the intermembrane space is ~25 fold higher than that in the matrix. The entry of protons into the cytoplasmic half-channel is also facilitated by a +0.14V membrane potential, which increases the proton concentration in the orifice of this channel. Protons entering the cytoplasmic half-channel reach Asp61. Protonation neutralizes this residue, which moves into the lipid bilayer, finally turning the rotor. However, throughout the whole rotation of the rotor, an Asp61 facing the matrix half-channel should be deprotonated thanks to the stator charge of Arg210 on subunit a (Figure 1). If both Asp61 sites facing half-channels are protonated at the same time, the rotor turns freely in both directions [3-7].

Proton conduction in the channels is proposed to occur via hopping and reorientation of protons (H^+^, not H_3_O^+^) – or deuterons if heavy water is substituted for water – by a Grotthuss or proton wire mechanism, and has been shown to be subject to an isotope effect [8].

## Theory and Discussion

### Deuteronation of ATP synthase

There is a pKa difference of 0.35 (difference between pH and pD) between protonated and deuteronated Asp61. This increase of pKa probably slows the dissociation of the deuteron. The stator charge of Arg210 is normally sufficient to ensure the dissociation of the Asp61 facing the matrix half-channel. However, this dissociation is likely to be slower at this new pKa, temporarily causing free movement of the rotor in both directions [3,4][9,10]. Nevertheless, it is not expected that deuteronation will completely inhibit ATP synthase activity: if this were the case, given a maximum proton transit rate through F_0 _of ~1000/s [11], we would expect ATP synthase activity to stop at the probable passage of deuterons within ~15 seconds. Since the half life of the c subunit of F_0 _is 40–50 hours [12], this would kill an organism within a very short time.

Molecular motors, unlike normal motors, are subject to thermal fluctuations (Brownian motion) [13]. The time that deuteron on Asp61 faces the matrix half-channel (while not inside the lipid bilayer) may not be sufficient for dissociation to be complete as fast as proton, since it dissociates more slowly than a proton. This can be tested *in silico *by molecular dynamics simulation studies on 3 dimensional atomistic models of F_0 _in water. In the models ~1:6600 ratio of deuterium to hydrogen and ~1:15000 ratio of deuteron to proton must be secured.

It has been shown experimentally that the kinetics of the F_1 _(ATPase) part of ATP synthase do not change in the presence or absence of D_2_O [14]. However, the kinetics of F_0 _rotation in D_2_O were not examined.

### Biological effects of deuteronation

The dissociation of a deuteron from Asp61 of the c subunit when exposed to the matrix half-channel is likely to be slowed, since it is exposed to the channel for a very short time because of the Brownian motion of the c subunit. This slow dissociation may cause temporary stutter in the rotor. If we were able to observe all ~15000 ATP synthases (Table 1) in a mitochondrion, we would see a percentage of them stuttering at any given time.

The deuteronation process may also disturb the function of proton coupled electron transfer (PCET) reactions. For example, it is likely to slow down electron transport in the ETC in mitochondria and cause the upstream accumulation and leakage of electrons, leading to increased free radical generation.

Since the deuteron dissociates more slowly than the proton, protons on the water-exposed parts of macromolecules (e.g. DNA, RNA, proteins) can exchange with deuterons. A deuteron has twice the mass of a proton and it makes stronger and shorter bonds with different bond angles [15,16]. The likely increase of deuterons on the water-exposed parts of macromolecules (especially ones having long half-lives) over time may cause conformational changes in a stochastic manner. These changes, if they occur in the active sites of enzymes, are likely to affect enzymatic activities.

There are many studies showing that different types of macromolecules can be affected by H/D exchange. Kinetic solvent isotope effects (KSIEs) represent the effect of isotope (e.g. H/D) exchange on the rate constants of enzymes. The activity of hepatitis delta virus (HDV) ribozyme was reported to show significant KSIEs [17]. A four fold decrease was reported in proton permeability coefficient of chloroplast lipid bilayers when H_2_O was replaced with D_2_O [18]. Solvent and substrate isotope effect on the activity of 4-methoxybenzoate monooxygenase from *Pseudomonas putida *was reported [19]. An effect of nucleotide binding on H/D exchange was reported in ATP synthase from the thermophilic *Bacillus PS3 *[20]. The difference in protonation between the native and unmodified forms of *Escherichia coli *tRNA(val) was shown by H/D exhange in NMR studies [21].

## Conclusion

If we consider the very high concentration of water in biological systems, it becomes difficult to neglect the relative amounts of deuterated water and deuterons. It seems likely that deuteronation of ATP synthase and other macromolecules has stochastic biological consequences. The proposed mechanism could shed light on the mechanisms of heavy water toxicity and on certain time dependent pathological processes such as aging. The change in the deuteronation level of purified macromolecules can be measured in physiological/pathological processes by Elemental Analysis coupled with Isotope Ratio Mass Spectrometry (EA-IRMS). Specific deuteronated positions on the molecules can be characterized by NMR spectroscopy studies. The effects of deuteronation, if any, can be delayed or prevented by decreasing the intake of deuterated water or increasing the turnover of organelles and macromolecules by stimulating autophagy-like mechanisms.

## Competing interests

The author(s) declare that they have no competing interests.
